# The Ethical Past of the Man at the Helm

**Published:** 1909-02

**Authors:** 


THE
### TEXAS MEDICAL JOURNAL.
Established July, 1885.

F. E. DANIEL, M. D., - - - - Editor, Publisher and Proprietor.

PUBLISHED MONTHLY.—SUBSCRIPTION $1.00 A YEAR.

VOL. XXIV. AUSTIN, FEBRUARY, 1909. No. 8.

The publisher is not responsible for the views of contributors.

### Original Articles.

For Texas Medical Journal.

### The Ethical Past of the Man at the Helm.

AN ARTICLE BY THE EDITOR OF THE JOURNAL OF THE A. M. A., SECRETARY OF THE A. M. A., THE GREATEST ARTIST IN MEDICAL "FLIP FLAP" THE WORLD HAS EVER SEEN, WITH COMMENTS BY G. FRANK LYDSTON, M. D., CHICAGO.

### MEDICAL ETHICS.*

Dr. Geo. H. Simmons wrote: Editor *Medical Brief*.*—Medical ethics has finally "struck" the *Brief;* and so, of course, it will now be thoroughly cussed and discussed. Dr. Nicholson, in the February *Brief,* does not show *quite as much bigotry as some members of the allopathic school. Now, I am a homeopath, and take several allopathic journals, as well as those of our own school. I have also taken a course in a "regular" college; and so I think I know as well as 99 out of every 100 old-school doctors what to do in a given case, according to the teachings of their school.* And at the same time I would just remark, in parenthesis, that not one allopath in 500 would prescribe homeopathically in a given case. And this is about the gist of the matter: *Those who run homeopathy down most known least about it.*

But I am afraid I am "off the question." Why should not allopaths counsel with homeopaths and eclectics? Our friend Dr. Nicholson takes Dr. Carpenter to task about those mercury and

**Medical Brief*, April, 1883, p. 168.

chalk powders, and intimates that the homeopath was the one who did the bungling, and then asks how Dr. C. would have felt if it had been on the other side, and the homeopath had suggested the powders. Would he (Dr. C.) have debased himself so much as to permit the putting up of the *"billionth of one millionth dose of hyd-cum-creta?* Yes, there it is. *It is the dose part that hurts.* Allow me to inform Dr. Nicholson that the dose has nothing to do with homeopathy, strictly speaking. When Hahnemann first promulgated the law of similars he did so from the effects which he observed from the use of Peruvian bark. The minimum dose was an after-proving. The law which homeopaths claim to follow is "similia similibus curantur"—nothing more. *We believe in giving the smallest particle of medicine that will have an effect to cure. The practice of the allopath is to give as much as the patient can stand.*

And yet the very next article in the *Brief* is entitled "Parvules." Just fancy the "regular" of twenty years ago giving the fortieth grain of podophyllin at a dose. What has caused this wonderful change? Not homeopathy, of course.

If Dr. Brown, allopath, calls me, a homeopath, in counsel, he does it for one of two reasons—to satisfy himself as to diagnosis. If the former, he tells me, after we have examined the patient, that he is afraid he has done all he can, and asks me if I would suggest anything. If I think any homeopath remedy will help I say so, and, if there is any manhood about him, he will try it. Is he less a physician and a gentleman for that? He may not believe in my treatment, but he does not know it will not cure. He knows he has failed; I can but do the same. But if the second is the reason I am called, to get my views on diagnosis, is there any objection to that, O ye infallible allopaths, even if there is no other doctor within twenty miles? We want to counsel sometimes when medical treatment is not necessarily to be discussed. Dr. Brown may have a lady patient suffering from uterine trouble—a tumor or some abnormal growth, or a case in general practice that he does not understand, or a fracture or dislocation that he needs help in; there are but two doctors in the village—he and I—shall he call me in counsel? No; although I have taken just the same and as thorough a course as he, except, possibly, in therapeutics. If he belongs to his State society he can not, because I am not a "regular." *Bah! I claim that the average homeopath or eclectic is just as honest, just as truthful, just as intelligent, is just as well versed in anatomy, physiology, diagnosis, pathology, surgery or*

*obstetrics as the average "regular."* No one but a bigoted and egotistical homeopath, eclectic or allopath, would claim that his school of practice was infallible and the other school without any benefit, and, consequently, humbugs. *I believe in homeopathy, and yet I do not claim but I may have a case that I can not cure, and yet an allopath or eclectic can.* But the allopath who refuses to counsel with me says by his actions: "I know it all—you know nothing; if I can not cure this case, I know you can not." Such ideas might have been excused 300 years ago but not now. Not many months ago I was practicing in a town where there was only two doctors—an allopath and myself. We counseled together often, privately and publicly; we had many surgical cases, and each helped the other; we counseled on diagnosis and on treatment. If he called me in, and I was satisfied that I could help him with a homeopathetic remedy, I would suggest it and it would be given, and vice versa. We did not counsel as often on treatment as on surgery and diagnosis, of course. This doctor was a member of a State society which instructed their delegates to St. Paul to vote against admitting the New York State society, because of further action in reference to the new code. Furthermore, one of the delegates from this State to the American, at St. Paul, voted to keep out New York, when he, himself, within a month from that counseled with me, and suggested to another homeopath that he would like to assist him in any gynecological case he might have, as he intended to make that a specialty. This may be called consistency, but if so it is a new kind. Dr. N. thinks that a homeopath and an allopath can not be honest in their belief and counsel together. Absurd! If I call in counsel an allopath, not as to diagnosis, but as to treatment, I do so at the request or with the consent of the friends of my patient. They understand the general treatment that will be followed, and yet they do not desire me to give up the case. In one sense there might be no counsel; I simply ask the allopath to come in and give something, if he can suggest anything. I may not believe in large doses, and yet acknowledge that they do good sometimes, and as I can do nothing perhaps he can. I am not giving up my belief, nor am I inconsistent. But suppose we can not counsel constantly with reference to treatment; suppose we admit that much for the sake of argument; shall we not be permitted to counsel in a case of surgery, or obstetrics, or diagnosis? Would there be anything absurd in that? If so, I fail to see it. Yet no allopath who belongs to the State society, outside of New York can do so. *Why has the old school been so bitter against the homeopaths and eclectics? If it is not because you want to kill us out, what is the reason?* And yet homeopathy was never growing as rapidly as now. *We do not take the scum of the earth as our patrons, nor the ignorant. We ring the front door bell of the avenue and boulevards, and number among our patrons the educated, intelligent and wealthy.* For my part, I would rather have seen New York vote to readopt the old code last week than the action they did take. Gentlemen, the more you fight and try to put down homeopathy and eclecticism by such bigoted rules as you have made, the more it will flourish.

G. H. SIMMONS, M. D.

Lincoln, Nebraska.

[Italics mine.—G. FRANK LYDSTON.]

### COMMENTS BY G. FRANK LYDSTON, M. D.

Some of the features of Dr. Simmons' article are worthy of serious consideration:

Notwithstanding the decidedly illiterate quality and the suggestion of the kindergarten in the above delightful contribution to the literature of medical ethics, its erudite (sic) author was thirty-one years of age at the time it was written and, according to his own estimate of himself, was in the possession of a critical, absolutely balanced and perfectly crystallized judgment of all existing schools of medicine. He knew as much of regular (Simmonsology, "allopathic") medicine as was knowable, and was more than *au fait* in homeopathy. He neglected to mention that he once took a "course" in eclecticism, but we can take that for granted, as he claims to know all about that school of practice.

Here, then, was a mature man, claiming to be possessed of all that was needful in the way of knowledge of all schools of medicine and to be especially well versed in the comparative merits of homeopathy and regular medicine (Simmonsology, "allopathy"), who decided in favor of homeopathy. A few years later we find him at the helm of the official organ of the greatest scientifically regular medical organization in the world and secretary of the A. M. A.! By what process of mental "flip flap" did he reverse his medical creed? Logic would impel us to believe that his boasted judgment yielded to policy, and that a yearning for position and the fleshpots of Egypt overcame that mature, conscientious opinion which formerly decided in favor of homeopathy.

Alas! that so great and cock-sure a man should have repudiated for revenue and power only his first and only medical love.

And this man who so illiterately heralded his breadth and catholicity from the housetops, who preached a doctrine of therapeutic free love, is now chairman of the Council of Pharmacy of the A. M. A., which says: "Thou shalt not prescribe that which beareth not our hall mark, nor shalt thou even advertise the same in thy journal."

And such modesty! *"I am a homeopath and take* (sic) *several allopathic journals. I have also taken a course in a regular college; and so I think I know as well as 99 out of every 100 old school doctors what to do in a given case, according to the teaching of their school."*

Great Hippocrates! Revered Esculapius! Oh! modest Dr. Sweeney, awe-inspiring Munyon *et id genus omne!*

What were the names of those several "allopathic" journals? I have been practicing medicine thirty years and never yet have seen one of that kidney. Neither have ever I met an "allopath."

And were those journals all as scientific as the *Medical Brief*—a worthy enough journal according to its lights—which he has since repudiated as a message of the imps of outer darkness?

As I write I am reminded that when my friend Dr. Wyeth, of New York, publicly apologized in the *Journal of the A. M. A.* for the appearance of one of his articles in the *Medical Brief* some years ago. I wrote a letter to the *Journal* criticising Dr. Wyeth's position. *Simmons refused to publish my letter!* He was willing to hand his old friend the *Brief,* attacked in the *Journal,* but would not publish a word of fairness regarding it.

Dr. Wyeth, to whom I afterward sent a copy of the letter, informed me that he would have taken no exception to its publication in the *Journal.*

And how did Simmons "take" those "allopathic journals"—in powder, capsule, tabloid or fluid mixture? I suspect he in all consistency took them homeopathically. "By their works shall ye know them." Why didn't he read them?

And now for that "course" in a "regular college." What sort of a college was it—agricultural, theologic, dramatic or shorthand? Could it have possibly been a medical college? If so, did he also take that homeopathically—as he did those "allopathic" (Simmonsology) journals?

I think I can answer the latter question. If he had taken a full course of instruction in a school of regular medicine at the time his article was written—which I'd be willing to wager he had not—he undoubtedly took it in a homeopathic dilution. Being an orthodox homeopath he could find more potency in a week's lectures than an "allopath" (Simmonsology) could in a six months' course. Verily, educational high potencies are great stuff! If he should ever be reproached for having secured a diploma "while you wait," both he and the diploma mill from which he procured it could make a touchdown for safety—"high potency student, high potency course."

*Diploma Buchaniensis!* Why, a fortnight's correspondence course would have served! Modesty! Ye gods! I wonder if there's any regular of high standing who can say that, after "taking" several medical journals and a single croton oil course in a medical school, he knows as much as 99 out of 100 doctors of his own or any other school. Alas! for thy rhodomontades, *O Monsieur Le Capitaine Fracasse!*

Speaking of wagers, I think it would be a safe bet that George H. Simmons, A. M., M. D., L. M., LL. D., never in his life took a full course in a school of regular medicine.

"Those who run homeopathy down know least about it," quoth our master in medicine: His English is "rotten," but we will try and comprehend its meaning: (Northwestern University, please pardon the criticism of its LL. D.).

At first glance one might infer that he himself was disclaiming a knowledge of homeopathy, for he seems to have "run it down" like a sleuth hound. What he really means is, that those who most severely criticise homeopathy know least about it. If homeopathy had never had better defendants than Geo. H. Simmons, A. M., M. D., L. M., LL. D., 'twould have died a-bornin'. He claims to be equally *au fait* in both schools. Logic compels us to infer that he had given equal time and labor to each. Therefore, his assumption of the role of a homeopathic perfecto was based upon his "taking" several homeopathic journals and "a course" in a homeopathic school.

Geo. H. Simmons, A. M., L. M., M. D., LL. D., A. M. A. Rex, said that the practice of the "allopath" (Simmonsology) was to give *"as much medicine as the patient could stand."* Listen to the words of wisdom as they fall from Pato's lips!

When Simmons wrote that he must have been half asleep—and the half of him in which he carried the phantasmagoric thing he called his conscience was the part that slept.

Say, Simmons, what were the names of those journals you

"took," anyhow? In what "regular" college did you take that "course"? and of what did you "take" a "course"? Whatever the material was, it seems to have had an intellectually constipating effect.

Come to think of it, my dear sir, you were wise in swallowing it. The parturition of an idea is painful and, in some persons, the cranial cavity and the fourth section of the *primae viae* are homologous—being equally jejune.

When Geo. H. Simmons, A. M., M. D., L. M., LL. D., wrote the lines just quoted he "proved" (Simmonsology) himself either an ignoramus or a deliberate prevaricator—or both.

And now as to his profound knowledge of homeopathy: He claimed *en effet*, that the single law of homeopathy was *similia similibus curantur.*

Shade of Hahnemann! What about the doctrine of "high potencies" and of symptomatic therapy? Wonder if the learned A. M., L. M., M. D., LL. D., knew that wise old Father Hahnemann borrowed the law of similars from the holy therapeutic trinity of Hippocrates.

Would George H. Simmons, A. M., L. M., M. D., LL. D., dare assert in 1908 that in 1883 *the average homeopath or eclectic was just as well versed in anatomy, physiology, diagnosis, pathology, surgery or obstetrics as the average regular?"* This, mind you, has naught to do with latter day standards of either school—standards forced upon both State boards which, in their inception, at least, were regulars.

The doughty, omniscient Simmons, A. M., L. M., M. D., LL. D., A. M. A. Rex, carries his modesty to extremes. He asserts that "allopaths" (Simmonsology) were wont to call him in to settle a diagnosis—whether to "settle the patient's hash" deponent sayeth not.

"Great was Confucius
Before him there was no Confucius
After him there will be no Confucius
Great was Confucius."

And now for the milk in the cocoanut. The elevation of his caudal plumage is due to the fact that he once practiced in a town where there were *only two doctors*, an "allopath" (Simmonsology) and himself. What was the other poor fellow to do? Simmons rather had him between Scylla and Charybdis—between the whims of the laity and the prickings of conscience.

It is safe to say, O Leftenant Simmons, U. S. A., A. M., M. D., L. M., LL. D., that there was no alternative for the poor "allopath (Simmonsology) *"if there was no other doctor within twenty miles."* As to the "harm," that's another question—one which I could not answer without a comparative study of the mortality statistics of that afflicted town and a knowledge of the medical acumen of that one lonely "allopath" (Simmonsology).

As for the one time credo of Simmons, A. M., L. M., M. D., LL. D., *"I believe in homeopathy,"* no one will dispute that he believed in it. Whenever he changed location, as he often did, he was quite as ready to believe in anything else—providing it paid. Now, there was his secular advertising stunt, for instance, and his latter-day saintly repudiation of his former creeds and associates—but, nuff said:

The leopard may not change his spots but the poor beast was never an A. M. nor an L. M. nor an M. D. nor an LL. D. nor a great editor, nor secretary of a great medical association. Neither was a salary of $10,000 a year and trimmings ever dangled in front of the leopard's nose.

Note the humane, philanthropic spirit of George H. Simmons, A. M., M. D., L. M., LL. D., A. M. A. Rex:

*"We do not take the scum of the earth as our patrons, nor the ignorant. We ring the front door bell of the avenue and the boulevards and number among our patrons the educated, intelligent and wealthy."*

Evidently his patrons were not so particular as he in the matter of education and intelligence. Evidently, also, his bump of acquisitiveness is no new development. And only one bell to all the boulevards and avenues, and Simmons had it!

How Christ-like this illiterate, ineffable snob from Merrie England; and what a stigma this man tried to put on the homeopath! But it is incredible that anybody believed the worshipper of the fleshpots—save in so far as his own ethics was concerned. The true physician, of whatever creed, has ever stood ready to relieve the ills of the poor and ignorant—aye, even of the "scum of the earth."

"Why was the old school so bitter?" etc. Oh, why, indeed? Let me whisper to thee, O peerless medical apostate from the sagebrush; simply because irregular medicine was infested with such half-baked, illiterate specimens as yourself, O mighty editor, A. M., M. D., L. M., LL. D.—men who were no ornament to the school they so badly represented.

I would I might further criticise the epistolary lucubrations of the greatest modern exponent of scientific flip flap. But one must yield to his own limitations in the matter of patience. Bad English and worse grammar doth weary me to my soul's marrow, and I would not harrow the bones of poor old Lindley Murray.

I would respectfully refer Simmons' wonderful document to the LL. D. department of Northwestern University for further elucidation and higher criticism.

---

For Texas Medical Journal.

